# Transplantation of mesenchymal stem cells reverses behavioural deficits and impaired neurogenesis caused by prenatal exposure to valproic acid

**DOI:** 10.18632/oncotarget.15245

**Published:** 2017-02-09

**Authors:** Nikolai Gobshtis, Matanel Tfilin, Marina Wolfson, Vadim E. Fraifeld, Gadi Turgeman

**Affiliations:** ^1^ Departments of Pre-Medical Studies & Molecular Biology, Ariel University, Ariel, Israel; ^2^ The Shraga Segal Department of Microbiology, Immunology and Genetics, Center for Multidisciplinary Research on Aging, Ben-Gurion University of the Negev, Beer-Sheva, Israe

**Keywords:** hippocampal neurogenesis, mesenchymal stem cells (MSC), doublecortin (DCX), behavioural disorders, valproic acid (VPA), Gerotarget

## Abstract

Neurodevelopmental impairment can affect lifelong brain functions such as cognitive and social behaviour, and may contribute to aging-related changes of these functions. In the present study, we hypothesized that bone marrow-derived mesenchymal stem cells (MSC) administration may repair neurodevelopmental behavioural deficits by modulating adult hippocampal neurogenesis. Indeed, postnatal intracerebral transplantation of MSC has restored cognitive and social behaviour in mice prenatally exposed to valproic acid (VPA). MSC transplantation also restored post-developmental hippocampal neurogenesis, which was impaired in VPA-exposed mice displaying delayed differentiation and maturation of newly formed neurons in the granular cell layer of the dentate gyrus. Importantly, a statistically significant correlation was found between neuronal differentiation scores and behavioural scores, suggesting a mechanistic relation between the two. We thus conclude that post-developmental MSC administration can overcome prenatal neurodevelopmental deficits and restore cognitive and social behaviours via modulation of hippocampal adult neurogenesis.

## INTRODUCTION

A growing body of evidence links neurodevelopmental changes, in neurogenesis in particular, with lifelong changes in cognition and behaviour [1, 2]. As a result, neurodevelopmental impairment not only trajects on future neural functions but may also share pathological mechanisms with age-related neurobehavioral disorders [3, 4]. Understanding these mechanisms and applying proper interventions may have implications not only for the neurodevelopmental disorders *per se* but for the maintenance of lifelong mental health as well. With regard to the above, a principal question arises as to whether neurodevelopmental deficits can be reversed later in life.

Neurogenesis has a critical role both in brain development and brain aging and has been suggested as a promising target for correction of neurobehavioral deficits [3, 5, 6]. In attempt to correct the impaired neurogenesis and associated pathologies, the transplantation of mesenchymal stem cells (MSC) has been proposed and tested in several models [7]. Bone marrow-derived MSC are multipotent stem cells able to differentiate to various mesenchymal lineages [8, 9]. Moreover, MSC are known for their neurogenic potential and can promote neurogenesis *in vitro* and *in vivo* [7]. We and others have shown that intracerebral transplantation of MSC can increase adult hippocampal neurogenesis and improve the related impaired behaviour such as cognitive, social and depressive-like behaviours [10–15].

Recently, it was shown that the prenatal exposure of rodents to the valproic acid (VPA, a known antiepileptic drug), though increasing neurogenesis in the cortex during development, resulted in a long-term reduction in adult hippocampal neurogenesis [16], accompanied by prominent behavioural deficits including cognitive and social behaviours [17, 18]. It was also shown that physical activity could partially restore neurogenesis and cognitive deficits caused by the developmental exposure to VPA [16].

Here, we postulated that (i) post-development transplantation of MSC may restore neurobehavioral deficits caused by prenatal (developmental) exposure to VPA, and (ii) the transplanted MSC would likely improve the adult hippocampal neurogenesis, as a putative underlying mechanism for the MSC therapeutic action.

## RESULTS

### Transplantation of mesenchymal stem cells improves behavioural deficits caused by prenatal VPA exposure

**Figure 1:**
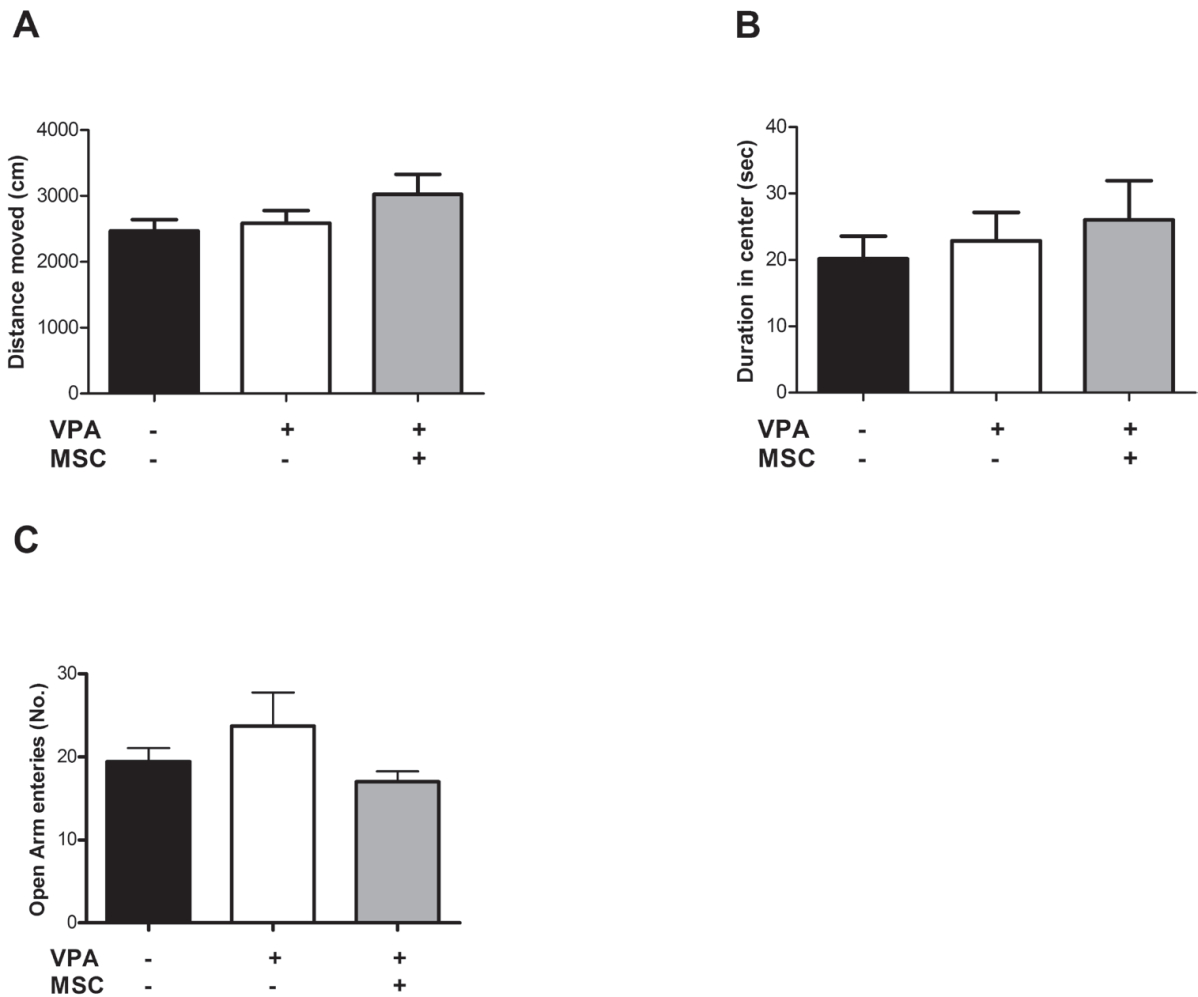
Open field and elevated plus maze assays did not reveal differences between VPA-exposed and untreated mice Open field assay did not show significant differences between VPA-treated and untreated mice, transplanted or not with MSC at the age of 7 weeks. Both general locomotion (total distance moved) and duration in the center of the arena were unchanged (**A** and **B**, respectively). In the elevated plus maze assay, (**C**) no differences in the number of entries to the open arms were observed. Data presented in the graph as means ± standard error.

Neither prenatal treatment of mice with VPA nor subsequent transplantation of MSC affected the general locomotor activity, as reflected by total walking distance in the open field test. As seen in Figure 1A, no significant differences between the two groups of VPA-treated mice (sham-operated and transplanted with MSC) as well as between these groups and untreated mice were found. Similarly, no significant differences between the three groups were observed in anxiety related behaviour assessed by the time spent in the center of the open field test (Figure 1B) and the number of entries to the open arms in the elevated plus maze test (Figure 1C).

**Figure 2:**
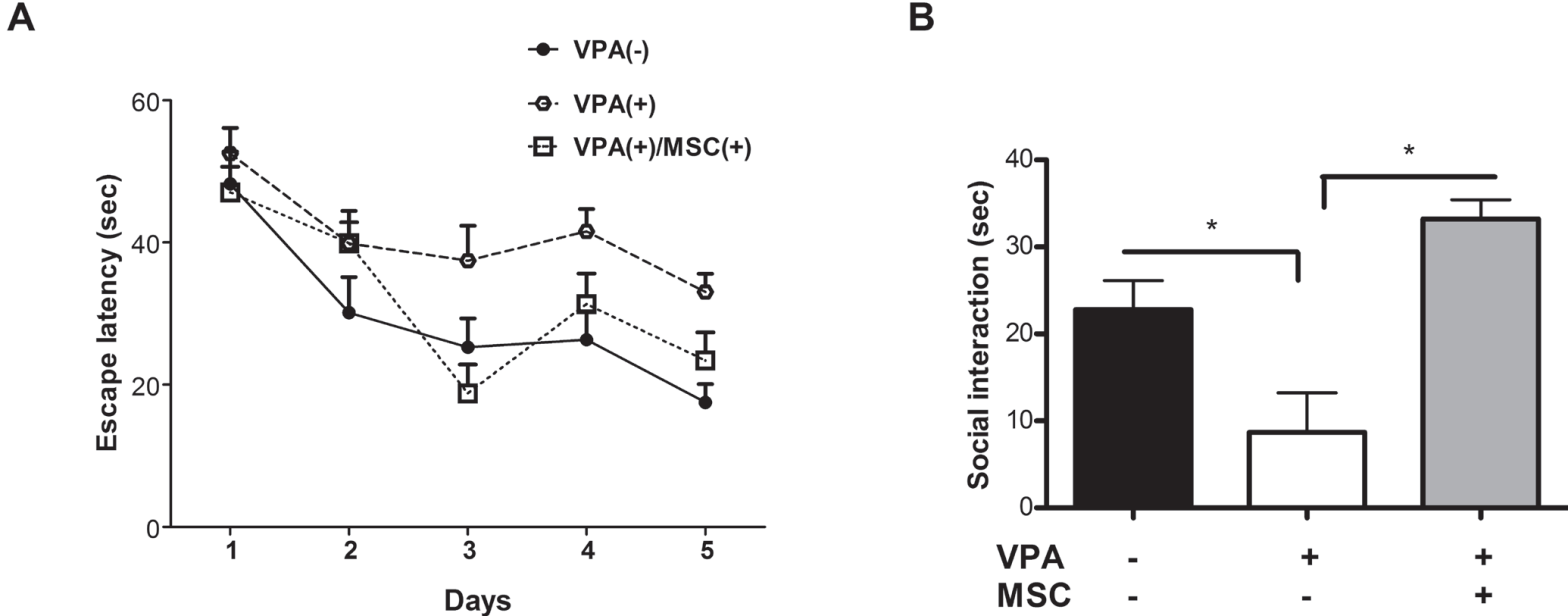
Mesenchymal stem cells transplantation can restore cognitive and social behaviour in VPA-treated mice **A**. 7-week-old mice were assayed for spatial learning in the MWM paradigm for 5 days. Escape latency was measured each day in 4 separate trials. The learning curve of VPA-treated mice significantly differed from that of untreated mice and MSC-transplanted mice (** *p* < 0.05, ANOVA repeated measures, *n* = 7). **B**. Sociability measured as the total duration of interaction between the tested mice with social stimuli in the three chambers assay showed a significant decrease (* *p* < 0.05, one-way ANOVA, *n* = 13) in VPA-treated mice compared with untreated and MSC-transplanted mice. Data presented as means ± standard error.

As opposed to the general locomotor activity, prenatal treatment with VPA had a significant impact on cognitive and social behaviours of mice (Figure 2). In the spatial learning assay (Morris Water Maze [MWM] test), the VPA-treated mice exhibited an impaired learning behaviour, as was evident by a longer escape latency durations on the third day onward of the trial compared with untreated mice (Figure 2A). Transplantation of MSC to VPA-treated mice almost fully restored the escape latency time which insignificantly differed from the untreated mice (Figure 2A). Similar effects were also observed in the sociability assay, where the untreated and MSC-transplanted VPA-treated mice spent significantly more time in interaction with social stimuli, compared with the VPA-treated mice that were not transplanted with MSC (Figure 2B).

### Transplantation of mesenchymal stem cells promotes maturation of newly formed neurons in the dentate gyrus of VPA-exposed mice

**Figure 3:**
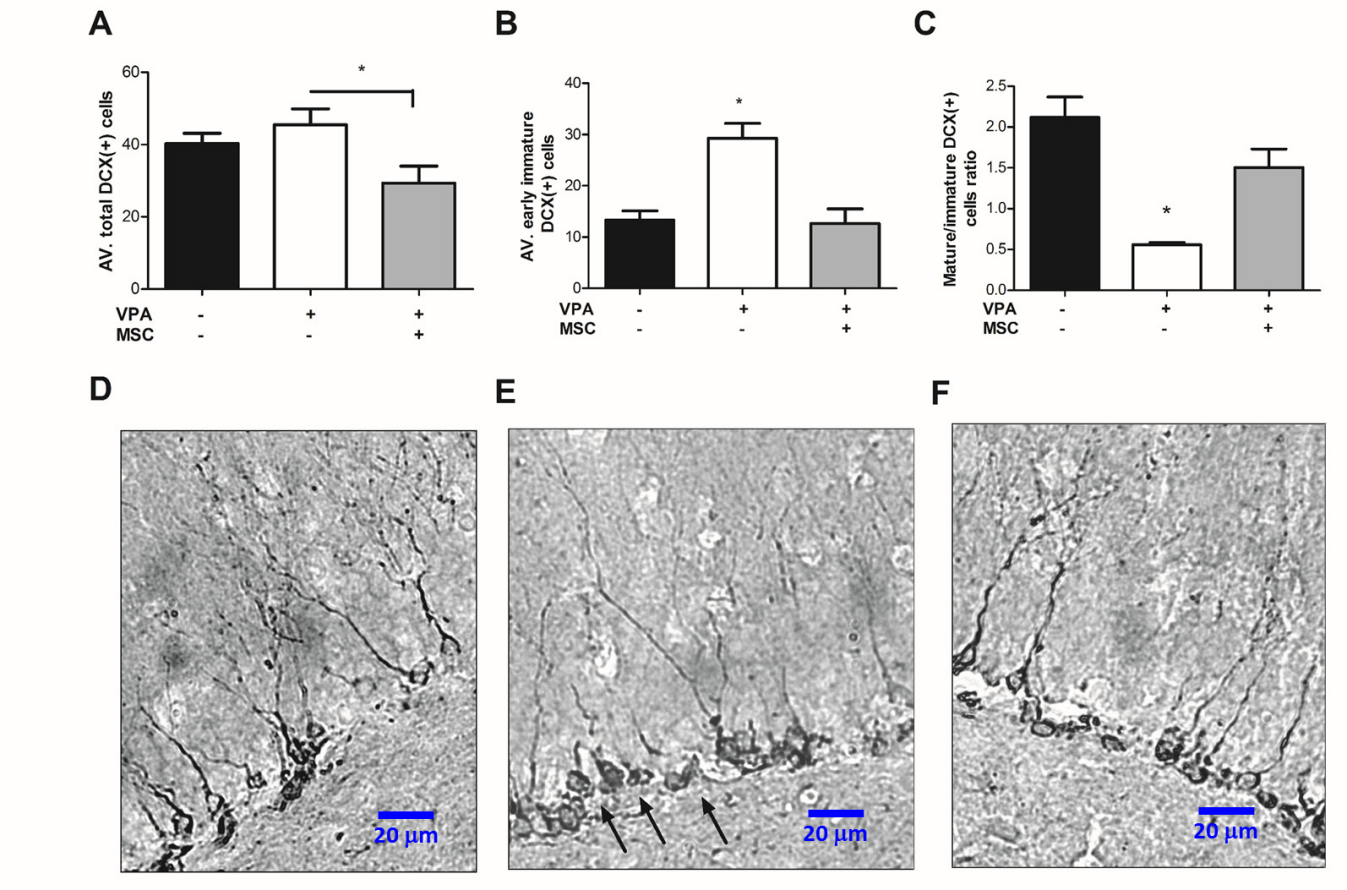
Mesenchymal stem cell transplantation can repair impaired hippocampal neurogenesis in VPA treated mice Frozen 20-micron brain sections were immune-stained for the expression of DCX in the granular cell layer of the DG. Positive DCX expression was differentially counted for immature, early differentiating cells (presenting no dendritic extension) and for maturing DCX-positive cells presenting dendritic extension. Average cell-number per hippocampus was calculated for each mouse, and data is presented in graphs as means ± standard error. **A**. Average number of total DCX(+) cells demonstrates a significant reduction in MSC-transplanted mice compared with non-transplanted VPA-treated mice (*p < 0.05, one way ANOVA, *n* = 4-7). **B**. Average number of immature DCX-positive cells is greatly increased in VPA-treated mice compared with untreated mice and MSC-transplanted mice (*p < 0.05, one-way ANOVA, *n* = 4-7). **C**. Average ratio of mature/immature DCX-positive cells was significantly reduced in VPA treated mice compared with the untreated and MSC transplanted mice (*p < 0.05, one-way ANOVA, *n* = 4-7). **D**., **E**. and **F**. micrographs of representing dental gyri of the untreated, VPA-treated and MSC- transplanted mice, respectively (mag. X400, scale bar 20 μm). Note the accumulation of immature DCX-positive cells (arrows) *versus* reduced number of maturing DCX-positive cells presenting dendritic branching, in VPA-treated mice (**E**) compared with the untreated (**D**) and MSC-transplanted mice (F).

Mice sacrificed at the age of 2 months were analysed for the formation of newly differentiating neurons in the dentate gyrus (DG) by immunostaining for doublecortin (DCX). Surprisingly, the total average number of DCX(+) cells in the DG was significantly lower in MSC-transplanted mice compared with non-transplanted VPA-treated mice (Figure 3A). However, when distinguishing between early differentiating DCX(+) cells (which present a round cell body with no dendritic extensions) and maturing DCX(+) cells (exhibiting dendritic extension), it could be noted that the VPA-treated mice had a significantly higher number of early immature DCX(+) cells than untreated or MSC-transplanted mice (Figure 3B, 3D-3F). As a result, the VPA-treated mice had a significantly lower ratio of maturing DCX(+) cells to early immature DCX(+) cells than untreated or MSC-transplanted mice (Figure 3C).

**Figure 4:**
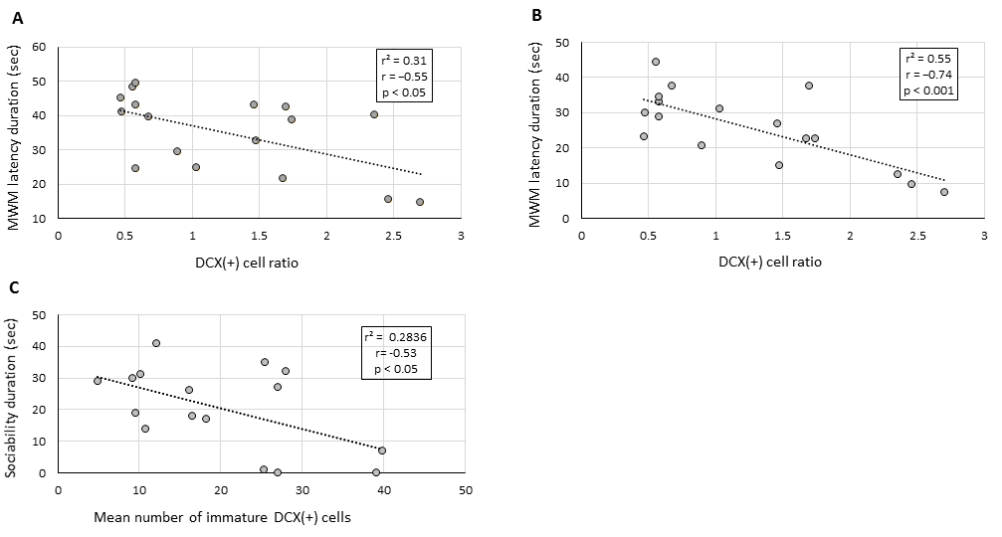
Deficits in cognitive and social behaviour correlate with hippocampal neurogenesis impairment Linear regression graphs depicting DCX(+) cell ratio between mature and immature DCX(+) neurons in the granular cell layer of the DG and the latency duration obtained by the same animals on day 4 and 5 of the MWM assay, (**A** and **B)**, respectively. (**C)** Linear regression graph depicting the average number of immature DCX(+) neurons in the granular cell layer and sociability duration of the same animals in the social interaction assay. The relationship between the variables was assessed by the Pearson's correlation coefficient.

In accordance with the behavioural tests, a significant inverse correlation was found between the DCX(+) cell ratio and the escape latencies in the MWM on day 4 and 5 of the assay (r = −0.55, *p* < 0.05 and r = −0.74, *p* < 0.001, respectively; Figure 4A and 4B, respectively). Similarly, an inverse correlation was observed between the average number of early immature DCX(+) cells and the duration of social interaction in the sociability assay (r = −0.53, *p* < 0.05; Figure 4C). These results indicate that accumulation of early differentiating DCX(+) cells *versus* mature DCX(+) cells correlates with a lower sociability and cognition as observed in the VPA-treated animals, thus suggesting that the observed behavioural changes may be mechanistically related to the changes in hippocampal neurogenesis.

## DISCUSSION

Prenatal exposure to VPA (a commonly used anti-epileptic drug) is a well-known risk factor for neurodevelopmental disorders in humans and a prominent model for autism in rodents [17–19]. Similarly to previous reports [17, 18], we observed clear cognitive and social deficits in mice prenatally exposed to VPA, whilst we did not observed significant changes in the level of anxiety as occasionally reported in the literature [20]. Comparison of behavioural changes in young mice caused by prenatal exposure to VPA with reported age-related changes showed that they have much in common. Indeed, complex learning tasks, such as the MWM test for spatial learning and memory, appear to be the most sensitive to age-related changes [21], and a decline in cognitive functions is well described in aging [22, 23]. Social interaction also decreases with advanced age, whereas less consistent results were reported for the impact of aging on anxiety-like behaviour [22, 24]. Thus, young mice prenatally exposed to VPA display some characteristic phenotypic features of brain aging and, to some extent, could serve as a model of accelerated aging of cognitive and social functions.

Prenatal neurobehavioral teratogenicity presents a great challenge to overcome. Cell therapy has long been a promising avenue in this regard. Early studies with neural grafting of fetal tissues have demonstrated for the first time the potential of cell therapy in reversing behavioural defects in adulthood, induced *during* pregnancy [25]. Since then, the evolvement of stem cell therapy has shown a similar promise, using stem cells from different sources [26, 27]. Yet, while the capability of transplanted stem cells to correct *postnatally* induced neurobehavioral deficits has been extensively explored, the therapeutic potential of stem cells to repair the adult manifestations of *prenatal* neurodevelopmental disorders has been addressed to a much lesser extent.

As we expected, MSC treatment of VPA-exposed mice repaired the VPA-induced behavioural deficits (Figure 2). Such potentially therapeutic effect of MSC on similar behavioural traits has been reported in the BTBR genetic mice model for autism and even in humans [13, 28]. Similarly, we have previously shown that transplantation of MSC (neural stem cells) can restore neurobehavioral deficits caused by developmental exposure to organophosphates [29]. These studies clearly indicated that, following stem cell treatment, the changes in neurogenesis coincide well with behavioural changes. Yet, an important point is that the engrafted MSC *do not* differentiate to new neurons but rather *paracrinely* support endogenous neurogenesis [12, 13, 30]. Indeed, in previous studies we observed that MSC injected to the lateral ventricle engraft adjacent to neurogenic niche sites including hippocampal molecular layer, DG hilus and ventricle choroid plexus, without any signs of differentiation [12, 31].

While the pathophysiology of VPA-induced neurobehavioral disorders is still unknown, several animal models have drawn attention to possible cellular, genetic and molecular mechanisms involved [32–34]. Specifically, Juliandi et al. (2015) reported that prenatal exposure to VPA increased embryonal cortical neurogenesis but decreased a long-term adult hippocampal neurogenesis [16]. They also reported on aberrant morphological characteristics of DG DCX(+) neurons. In the present study, we assessed the rate of hippocampal neurogenesis by quantifying the number of DCX-expressing cells in the DG, i.e. the cells representing newly formed differentiating neurons [35, 36]. Surprisingly, we found no significant difference in the total number of DCX(+) cells between VPA-treated and untreated mice (Figure 3A). Moreover, the MSC transplantation to VPA-treated mice significantly decreased the total number of DCX(+) cells in the DG as compared to VPA-treated and untreated groups. However, we noted a prominent number of early differentiating DCX(+) neurons and a relatively low number of mature DCX(+) neurons in the DG of VPA-treated animals. The opposite results were found in untreated mice and MSC-transplanted mice exposed to VPA (Figure 3). This observation was statistically confirmed by calculating the ratio of early differentiating cells to mature DCX(+) cells in the DG. Since DCX expression continues during neuronal differentiation [37], we presume that the accumulation of early differentiating/immature DCX(+) neurons in the DG of VPA-exposed animals is a result of their failure to continue differentiation. A similar concern was raised by Juliandi et al. (2015) [16]. Moreover, here we report on statistically significant inverse correlation between accumulation of immature DCX(+) neurons and both cognitive and social behavioural performance (Figure 4). These findings suggest that impaired neurogenesis observed in VPA-treated mice is mechanistically related to the behavioural deficits caused by exposure to VPA, and a reversal of both by MSC transplantation. Remarkably, Kuipers et al. (2015) reported that increasing age significantly reduced the number of mature DCX(+) cells and, consequently, increased the fraction of immature DCX(+) cells in the mouse hippocampus [6]. This further supports the idea that prenatal exposure to VPA may serve as a model of accelerated brain aging.

Among the suspected teratogenic effects of VPA is the inhibition of histone deacetylase enzymes (HDAC) activity [17]. This inhibition results in a marked increase in histone acetylation, DNA demethylation, and ultimately alters the global gene expression in the central nervous system [16, 38]. These epigenetic changes can be inherited to daughter cells, persist for long term and some concerns have been raised for their inheritance to offspring as well [2]. Neuronal development is governed by corresponding epigenetic changes, i.e. by histone modification and DNA methylations and demethylations [39, 40]. It is therefore conceivable to assume that exposure to VPA at critical developmental time points may impose epigenetic changes that will hamper future differentiation of the daughter cells and impair neurogenesis even later in life. Impaired neurogenesis may in turn inflict neurodevelopmental disorders or await for additional (age-related) factors causing further deterioration in hippocampal neurogenesis or challenging impaired neurogenesis to result in behavioural deficits. Indeed, both age-related decline in cognitive functions and neurodegenerative diseases are associated with reduced hippocampal neurogenesis [41]. Furthermore, based on recent evidence, a link between developmental neuroplasticity and neurodegenerative processes later in life has been suggested [5]. Thus, the study of neurodevelopmental disorders may have implications on the understanding of aging-related conditions and their prevention as well. The prenatal VPA exposure model for neurodevelopmental impairment is a valid model in this regard, having long-term effects extending to adulthood [42]. It is both relatively low-cost and therapeutic studies can yield results in a relatively short term compared with standard aging models.

As we report in this study, post-developmental intracerebral administration of MSC can reverse the observed changes in neurogenesis and behaviour, caused by prenatal exposure to VPA. Whether this intervention also reverses the putative epigenetic changes inflicted by VPA is yet an open question. A positive answer may stress the importance of treating neurodevelopmental impairments for the maintenance of lifelong cognitive function and minimizing the age-related deterioration in cognition. In our opinion, MSC or their products can potentially provide an efficient means in achieving this goal.

## MATERIALS AND METHODS

### Animals

All experimental procedures were approved by Ariel University Animal Care and Use Committee and were done in accordance with National Institutes of Health guidelines. Three-month-old female ICR mice were purchased from the Harlan Company (Jerusalem, Israel) and housed at the Ariel University animal facility under conditions of constant temperature (22 °C) on a 12:12 h light:dark cycle. Food and water were provided *ad libitum*. Female mice were bred with sexually experienced 5-6-month-old male ICR mice. At embryonic day 12.5, pregnant females were injected intraperitoneally (ip) with VPA (cat. no. 1069-66-5, Sigma, Israel) at a dose of 500 mg/kg. Control pregnant mice were injected with a vehicle (saline 0.9%). Offsprings were housed under the same conditions and male offspring were used for the experiments.

### Isolation, characterization and transplantation of mesenchymal stem cells

**Figure 5:**
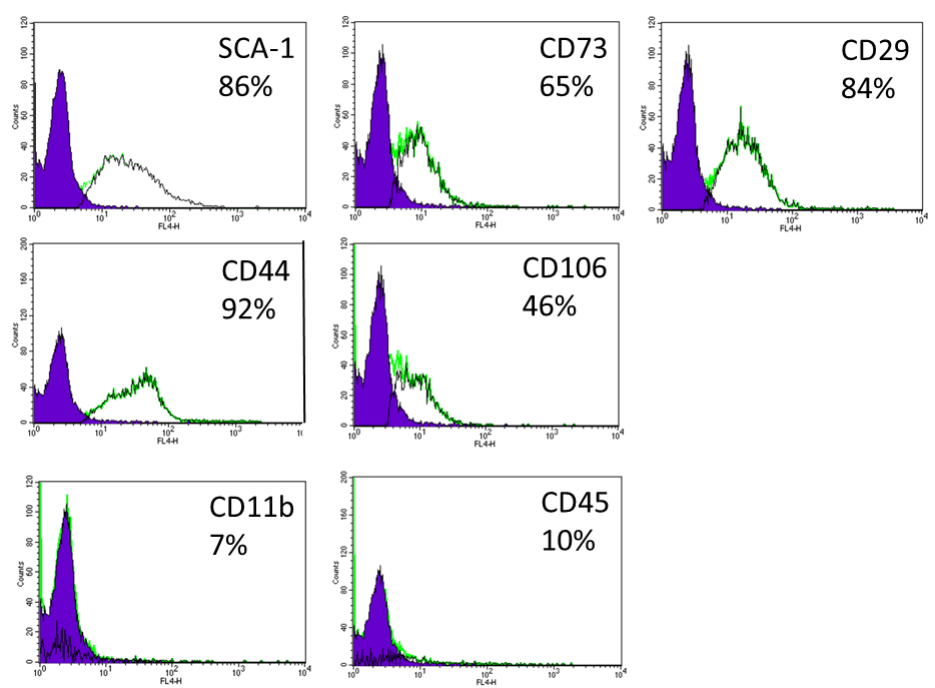
Immunophenotype of MSC cultures Cultured MSC were stained with fluorescently labelled antibodies for mesenchymal (CD29, CD44, CD73, SCA-1, CD106) and hematopoietic markers (CD45, CD11b) using the mouse multipotent mesenchymal stromal cell marker antibody panel (cat No. SC018; R&D systems; Minneapolis; USA). Negative control samples were processed similarly but without marker specific primary antibodies. The FACS analysis results are presented with the percent of positive cells for each marker. Filled histograms represent negative control distribution, and blank histograms represent MSC cultures stained with the appropriate marker specific antibody.

Mesenchymal stem cells (MSC) were isolated from the bone marrow of 2-month-old male ICR mice. Briefly, following the sacrifice of mice, the tibias and femurs were removed and cleaned from connective tissue. Marrow was flushed out of the epiphysis cut bones and suspended in Dulbecco's modified Eagle's medium (cat. No. 01-050-1A; Biological Industries, Beit Haemek, IL) supplemented with 10% fetal bovine serum (cat. No. 04-001-1A; Biological Industries), 100 units/ml penicillin (cat. No. 03-031-5C; Biological Industries), 100 μg/mL streptomycin (cat. No. 03-031-5C; Biological Industries) and 2 mM L-glutamine (cat. No. 03-020-1A Biological Industries). Suspended marrow cells were plated in a 100-mm^2^ dish and cultured at 37 °C in 95% air + 10% CO_2_ atmosphere, with removing the non-adherent cells 24 and 48 h following plating. Medium was changed twice weekly, and upon reaching confluence cells were sub-cultivated. MSC were expanded in culture for up to 20 passages. MSC cultures were immunophenotyped by FACS analysis (FACSCalibur with CellQuest software, Becton Dickinson, USA) for the widely used markers for MSC, using the mouse multipotent mesenchymal stromal cell marker antibody panel (cat No. SC018; R&D systems; Minneapolis; USA). MSC cultures were shown to be positive for the stromal markers CD29, CD44, CD73, CD106, SCA-1 and negative for the hematopoietic markers CD45 and CD11b (Figure 5).

VPA-exposed 5-week-old male offsprings were anesthetized by ip injection of ketamine (150 mg/kg, Ketaset, Fort Dodge, USA) and xylazine (10 mg/kg, Sedaxylan, Phibro, Israel). Anesthetized mice were stereotactically injected into the right lateral ventricle (coordinates referring to bregma: A = −0.058 cm, L = −0.11 cm, V = −0.3 cm) with either 10^5^/5 μl MSC or vehicle (saline), using stereotaxic apparatus (PWD Life Science Co, Ltd, China). At the age of 7 weeks, animals were subjected to a series of behavioural test.

### Behavioural assays

#### Open field test

The open field apparatus consisted of a black plastic box measuring 40 × 40 x 40 cm (width x length x height). The mice were placed near the midpoint of the open field apparatus for a period of 6 min. Using a computerized video system and software (EthoVision, Noldus; Wageningen, Netherlands), the total distance the animal walked and the time spent in the center of the arena were measured.

#### Elevated plus maze test

The elevated plus maze (EPM) test is widely used as a behavioural probe in rodents for evaluating an anxiety-like behaviour [43]. The EPM apparatus is comprised of two open arms (10 × 45 cm) and two enclosed arms (10 × 45 x 40 cm) extended from a central intersecting zone (10 × 10 cm). Mice were placed in the central area, facing one of the open arms, and were tested for 5 min. The trial was recorded using a computerized video system and software (EthoVision). The total number of entries to the open arms was recorded and reflected the level of anxiety (a higher number means lower anxiety).

#### Morris water maze test

The Morris Water Maze (MWM) test is widely used for evaluating spatial learning and memory in rodents [44]. The MWM arena is consisted of a round black plastic pool (diameter: 100 cm, height: 40 cm), filled with water to a height of approximately 25 cm, 1 cm above a hidden (escape) platform allocated in one point close to the pool's edge. Geometrical marks were placed on the pool's wall at an equal distance from each other. The temperature of the water was kept in the range of 23 ± 2 °C. The test was done for 5 consequent days: in each day each mouse was tried 4 times from different points in the arena for 1 min, with a time interval of 75 min between the trials. When the mouse reached the escape platform earlier than 1 min, swimming time was registered with the trial continued for 20 sec by allowing mouse to stay on the platform. If a mouse failed to escape during a swimming trial period, it was manually placed on the platform for the same time (20 sec). In each trial, the latency escape time taken for the mouse to find the hidden platform was recorded using EthoVision XT tracking system software.

#### Social interaction test

The social interaction test was based on the social recognition procedure described previously [45, 46]. Briefly, the social interaction apparatus was constructed in a form of a large open container (72 cm x 28 cm x 27 cm; width x length x height, respectively) divided into 3 equal chambers. In the two opposing chambers were placed two small wire cages (15 cm x 13 cm x 15 cm). At the first session, each tested mouse was placed in the empty apparatus and allowed to explore it and habituate for 20 min. In the second session of the test, a social stimuli (novel male mouse) was placed in one of the wire cages. Tested mice were allowed to explore the apparatus for additional 4 min. All trial sessions were monitored and analysed using EthoVision XT tracking system software. Total duration of interaction for each mouse with the social stimuli was recorded.

### Immunohistochemistry staining for doublecortin (DCX)

Following behavioural assays the animals were sacrificed by intracardial perfusion with 4% paraformaldehyde. Brains were removed and frozen brain tissue sections (20-μm) were prepared using MEV Slee Semi-Automatic Cryostat (SLEE medical GmbH, Germany). Hippocampal sections were stained for doublecortin (DCX) using immunohistochemistry kit according to the manufacturer's protocol (cat. No. AB2253; Millipore; Ma; USA). Briefly, sections were re-fixed with 4% paraformaldehyde for 10 min followed by 10 min incubation with 3% H_2_O_2_ for blocking endogenous peroxidase. Membranes were permeablized with 0.01% TritonX100 twice for 5 min and incubated in blocking solution (2% bovine serum albumin) for 40 min. Sections were then incubated with primary rabbit polyclonal anti-DCX (Abcam, #ab18723, diluted 1:1000 in 0.5% bovine serum albumin) overnight at 4 °C. Next, incubation with horseradish peroxidase (HRP) one-step polymer conjugated secondary antibody for 30 min at room temperature was commenced. Visualization was achieved with 5 min incubation with 3,3' Diaminobenzidine tetrahydrochloride (DAB) buffer with DAB chromogen (cat. No. #ACH500SkyTek lab, Utah, USA,). Between steps, sections were washed in Phosphate Buffered Saline for three times for 5 min. DCX-positive [DCX(+)] cells in the granular cell layer (GCL) of the dentate gyrus (DG) were counted under a microscope in 6 representing sections of the hippocampus and the average number of DCX(+) cells per DG per section was calculated for each mouse. Micrographs were acquired using using a Zeiss LSM 700 confocal microscope (Carl Zeiss, Germany) equipped with camera T-PMT and × 40 objective lens and processed with ZEN imaging software.

### Statistics

All Bar graphs and statistical analyses were prepared by GraphPad Prism 5 software. Regression plots were prepared using Microsoft Excel. Data are presented as mean ± standard error. The tests applied for evaluation of statistical significance were analysis of variance (ANOVA) followed by Tukey's post-hoc test. Pearson's test was applied for calculating correlations.

## Abbreviations

DCX, doublecortin; DG, dentate gyrus; EPM, elevated plus maze; GCL, granular cell layer;

icv, intracerebroventricular; ip, intraperitoneally; MSC, mesenchymal stem cells; MWM, Morris Water Maze; VPA, valproic acid

We thanks Dr. Hagai Yanai for his assistance in preparing the manuscript.
